# Intestinal short-chain fatty acid turnover is not associated with resting state functional connectivity in mesolimbic dopaminergic network in healthy adults

**DOI:** 10.1016/j.ynirp.2025.100285

**Published:** 2025-08-25

**Authors:** Madelief Wijdeveld, Anouk Schrantee, Júlia Tolra Azor, Francesca van Baarzel, Eelco van Duinkerken, Max Nieuwdorp, Richard G. Ijzerman

**Affiliations:** a Department of Internal and Experimental Vascular Medicine, Amsterdam University Medical Centers, location AMC, Amsterdam, the Netherlands; b Department of Radiology and Nuclear Medicine, Amsterdam University Medical Center, location AMC, Amsterdam, the Netherlands; c Department of Neurology, Universidade Federal do Estado do Rio de Janeiro, Rio de Janeiro, Brazil; d Department of Endocrinology, Amsterdam University Medical Centers, location VUMC, Amsterdam, the Netherlands

**Keywords:** Functional MRI, Resting state functional connectivity, Intestinal microbiota, Gut-brain axis, Short-chain fatty acids

## Abstract

People with obesity tend to have altered functional connectivity of reward-related areas in the brain, contributing to overeating and weight gain. The gut-brain axis may function as a mediating factor, with gut-derived short-chain fatty acids (SCFAs) as possible intermediates in the relationship between microbiota and functional connectivity. We investigated the influence of SCFA turnover on resting state functional connectivity in healthy individuals with extremely high and extremely low levels of intestinal SCFA turnover. In this study, we included individuals with low or high intestinal SCFA turnover (estimated by fecal concentration of the butyryl-coenzyme A (CoA)-transferase (ButCoA) gene). Resting state functional magnetic resonance imaging (rs-fMRI) was used to assess functional connectivity of eight regions of interest (ROIs) either directly involved in the mesolimbic dopaminergic network (amygdala, hippocampus, caudate nucleus, putamen and nucleus accumbens) or primary projection regions of this network (middle frontal gyrus, superior frontal gyrus, insula). Functional connectivity was assessed using connectivity strength and eigenvector centrality. No differences in connectivity strength or eigenvector centrality were observed between the high and the low ButCoA group in any of our ROIs, suggesting SCFA turnover is not associated with resting state functional connectivity of central reward-related areas. Although previous studies provide evidence for an association between gut microbiota and resting state functional connectivity of reward-related areas, our findings do not support the hypothesis that this relationship is mediated by SCFAs. This suggests the existence of alternative mechanisms via which the intestinal microbiota may affect appetite, beyond local SCFA production.

## 1 Introduction

Obesity is a complex condition caused by multiple factors including food abundance, systematic overeating and a sedentary lifestyle (Nishino et al., 2013). People with obesity tend to have altered signaling in the mesolimbic dopaminergic pathway of the brain, which is considered a major reward-related circuit (van Zessen et al., 2012). This pathway originates in the ventral tegmental area of the midbrain and extends to the nucleus accumbens (NAc), basal ganglia, the amygdala, and the hippocampus. Cortical projections from this network to the middle and superior frontal gyrus (MFG and SFG) and the insula cause a conscious hunger state that leads to food seeking at the appropriate time, but also induces a satiated state upon food intake (Eiselt et al., 2021; Wang et al., 2009). Central nervous system (CNS) dopamine signaling in general and mesolimbic signaling in particular are considered essential for the motivation towards food intake (Salamone et al., 2001). Previous studies have shown that obesity is associated with decreased dopaminergic signaling in the mesolimbic pathway and this leads to excessive eating as well as a tolerance to the rewarding effects of excessive eating (Wang et al., 2002, Kenny and Johnson, 2010).

Resting state functional MRI (rs-fMRI) analysis can provide insights into the communication and connectivity within intrinsic brain networks, such as the mesolimbic reward system, in the absence of a specific task (Fox and Raichle, 2007). Studies employing seed-based resting state connectivity analysis show that obesity is associated with increased functional connectivity between the hypothalamus and regions involved in reward and salience networks, but decreased connectivity between the hypothalamus and the prefrontal cortex and insula (Le et al., 2020; Lips et al., 2014). In contrast, the dorsal and ventral striatum and amygdala showed decreased connectivity with reward and salience networks, but increased connectivity with higher order processing regions (Gupta et al., 2018; Ding et al., 2020). Moreover, other studies have shown the caudate nucleus has a high nodal degree, whereas the insula and NAc have a low nodal degree in obesity, indicating the differential importance of these brain nodes in the whole-brain network of people with obesity (Meng et al., 2018; Park et al., 2018; Zhang et al., 2019). Using this technique, we have previously shown that the rate of resting state functional connectivity is linked to metabolic health, as it is associated to overweight in healthy female monozygotic twins (Doornweerd et al., 2017). In addition, we demonstrated that resting state functional connectivity is significantly affected in patients with longstanding type 1 diabetes (van Duinkerken et al., 2017).

Recent evidence suggests that the gut microbiota may play a key role in overeating and obesity (Lynch and Pedersen, 2016; Finnicum et al., 2018; Ridaura et al., 2013), in part through appetite modulation (Han et al., 2021). Recent clinical observational studies have applied rs-fMRI to study microbial effects on resting state connectivity of the brain (Curtis et al., 2019; Kohn et al., 2021). Results showed that higher microbial alpha and beta diversity was associated with stronger functional connectivity between the insula and several regions, including the frontal pole left, lateral occipital cortex right, lingual gyrus right and cerebellum (Curtis et al., 2019). Furthermore, abundance of specific bacterial genera was associated with network connectivity strength within the default mode, executive control and frontoparietal network (Kohn et al., 2021). However, it is so far unclear which mechanisms mediate the relation between microbiota and brain functional connectivity. In addition, considerable interindividual variability in these associations remains largely unexplored. Such variability may stem from host genetic differences, immune tone, dietary patterns, metabolic status, and lifestyle factors, which modulate the microbiota–brain axis in highly personalized ways. Moreover, differences in the functional capacity of microbial communities, including their ability to produce neuroactive compounds or influence systemic inflammation, could contribute to distinct connectivity patterns across individuals.

In this regard, intestinal short-chain fatty acids (SCFAs), derived from microbiota fermentation of dietary fiber, may function as important intermediates in the link between the intestines and appetite regulation. In human feces, the following SCFAs are generally detected: acetate, propionate, butyrate, valerate and caproate (Han et al., 2015). Of said SCFAs, acetate, propionate and butyrate are the most abundant in humans, and only acetate is taken up into the peripheral circulation in significant amounts (Chen et al., 2022). SCFAs influence host physiology through multiple pathways. They activate G-protein-coupled receptors (e.g. GPR41/43) on enteroendocrine cells, stimulating secretion of hormones such as peptide YY (PYY) and glucagon-like peptide-1 (GLP-1), which regulate satiety and glucose metabolism (Tolhurst et al., 2012; Lee et al., 2018). SCFAs can also bind to GPR41/43 receptors in the vagus nerve, which transmits this information to the central nervous system (CNS), where it is further integrated and distributed to appetite-regulating centers (Lal et al., 2001, Näslund and Hellström, 2007). In addition, specific SCFA acetate can cross the blood-brain barrier and in a study in mice, acetate has been shown to modulate the hypothalamic expression of genes related to appetite (Frost et al., 2014). SCFAs acetate and butyrate are considered to exert the most prominent effects on host satiety feedback mechanisms, in case of acetate via direct central effects, whereas butyrate is thought to exert appetite-suppressing effects via intestinal GLP-1 receptor activation and vagal nerve stimulation (Li et al., 2018). Some preliminary data in healthy subjects showed that increased microbial fermentation activity can significantly affect resting state functional connectivity (Bagga et al., 2019; Rode et al., 2022; Tillisch et al., 2013). All three studies were aimed at increasing intestinal fermentation activity, hereby increasing SCFA turnover (Pascale et al., 2018). Probiotic supplementation with SCFA-producing bacteria or fermentation product decreased resting state functional connectivity within the middle and superior frontal gyrus network and in the default mode network, but increased connectivity within the salience network (Bagga et al., 2019) and increased connectivity between the midbrain and the rest of the brain (Tillisch et al., 2013); however, another study using seed-to-voxel analysis showed conflicting results (Rode et al., 2022). These studies suggested increased intestinal fermenting activity as a mediating factor in the probiotic effects, however, none of these studies specifically address the role of SCFAs.

To breach this gap, we here aim to assess whether intestinal SCFA turnover is related to resting state functional connectivity of the reward-related circuitry by selecting two extreme groups in terms of SCFA turnover. This study comprises the secondary outcome of a larger study (Wijdeveld et al., 2023), in which we selected individuals from the highest or the lowest acetate and butyrate availability (estimated as the fecal concentration of the butyryl-coenzyme A (CoA)-transferase (ButCoA) gene (Diez-Gonzalez et al., 1999)) from the large HELIUS cohort (Snijder et al., 2017). As one of the main outcomes, we have tested group-differences (high ButCoA versus low ButCoA) in central regulation of appetite by means of task-based fMRI, but found no significant difference between the groups. Here, we used rs-fMRI to identify differences in functional connectivity between the high and the low ButCoA group. Due to the important role of the mesolimbic reward network in regulating appetite, we hypothesized that resting state functional connectivity of the mesolimbic pathway and its primary projection regions differs between high and low ButCoA individuals.

## 2 Materials and methods

### 2.1 Subjects and study design

This study comprises the secondary outcome of an observational study in 60 Dutch origin subjects who were recruited from Healthy Life in an Urban Setting (HELIUS) cohort study (Snijder et al., 2017), for additional data collection, aimed at examining the association of the intestinal microbial acetate and butyrate availability with insulin sensitivity, insulin secretion, and central appetite regulation (Wijdeveld et al., 2023). In the HELIUS cohort, at time of recruitment for the current study, qPCR was performed in 439 Dutch origin participants to measure the concentration of the ButCoA gene, as a measure of intestinal acetate availability (Diez-Gonzalez et al., 1999) (Supplementary Fig. S1). The ButCoA gene abundance was used as a proxy for high or low intestinal acetate into butyrate converting activity and therefore representative for SCFA turnover. See main article for complete fecal sequencing procedure (Wijdeveld et al., 2023). The 16S rRNA gene amplicon raw sequence data and associated metadata have been deposited at the European Genome-phenome Archive under accession code EGAD00001004106 (https://ega-archive.org/datasets). The HELIUS data are all owned by the Amsterdam UMC, location AMC in Amsterdam, the Netherlands. Any researcher can request the data by submitting a proposal as outlined at http://www.heliusstudy.nl/.

131 of the total 439 subjects either belonged to the highest 15 % or lowest 15 % intestinal ButCoA concentration, and those were approached by letter to participate in this study. Of those, 88 were willing to partake in the study. Upon rigorous screening (see below for in- and exclusion criteria), 60 subjects were included for this study (30 from the highest 15 % and 30 from the lowest 15 % of ButCoA concentration).

All participants were males or females >18 years of age (body mass index (BMI) 19–37.2 kg/m2). Exclusion criteria were: immunodeficiency, serious heart, liver, renal or neurological disease, malignancies, inflammatory bowel disease, diabetes mellitus type 1 or 2, pregnancy or breast feeding, MRI contra-indications, alcohol or drug abuse and current use of antibiotics or up to 3 months prior to the study visit. Inclusion criteria comprised age ranged 18–65, stable body weight (<5 % reported change during the previous 3 months) and Dutch origin. The study protocol was approved by the medical ethics committee of the Amsterdam University Medical Centre (approval code: NL65063.018.18) and performed in accordance with the Helsinki Declaration (version 2013). All participants provided written informed consent.

### 2.2 Anthropometrics and biochemistry

Participants were invited for a visit to the Amsterdam UMC for data collection for this current study. During their appointment, fasted blood samples were collected, followed by anthropometric body measurements and determination of body composition through bioelectrical impedance analysis (BIA) (BioScan 920-II; Maltron International Ltd; Essex, United Kingdom). The anthropometric measurements were used to calculate the subject's BMI, while body fat percentage was derived from the BIA. Blood was collected in Vacutainer tubes containing silica particles, heparin or Ethylenediaminetetraacetic acid (EDTA). The blood samples were centrifuged at 1550 g (4 °C, 15 min), after which the plasma and serum were stored (at −80 °C) until further analysis. A commercial assay on a Cobas 8000 c702 analyzer (Roche; Basel, Switzerland) was done to determine plasma glucose. Commercial assays (Diasys and WAKO) on the Selectra (Sopachem; Ochten, the Netherlands) were used according to the manufacturer's instructions to determine fasting plasma total cholesterol, HDL-C and triglycerides. The Friedewald formula was used to calculate LDL-C levels. Finally, ion exchange chromatography was used to determine HbA1c.

### 2.3 MRI image acquisition and processing

MRI data were acquired using a 3.0 T Elition scanner (Philips Healthcare, Best, The Netherlands) with a 32-channel receive-only head coil. 3D T1-weighted (T1w) structural imaging data were acquired using a magnetization prepared rapid acquisition gradient echo (MPRAGE) sequence. Hereafter, rs-fMRI scans were obtained. The rs-fMRI scans were conducted before the two fMRI tasks (Wijdeveld et al., 2023). Scans were performed with the following scan parameters: repetition time/echo time = 1500/30 ms; flip angle = 73°, slice thickness = 2.5 mm, field of view = 240 x 240 × 131.75 mm, resolution = 2.5 x 2.5 × 2.5 mm, multiband factor = 2, SENSE factor = 2, 400 volumes. MRI data were pre-processed using fMRIprep 20.0.6 (Esteban et al., 2019, 2020). In brief, each T1-weighted (T1w) scan was normalized to MNI space (Fonov et al., 2009). Pre-processing of functional data included motion correction (FLIRT) (Jenkinson et al., 2002), distortion correction (3dQwarp) (Cox and Hyde, 1997), followed by co-registration to the T1w image using FLIRT (FSL 5.0.9). Independent component analysis (ICA) based on Automatic Removal Of Motion Artifacts (AROMA) was used to generate data that was non-aggressively denoised (Gratton et al., 2020), and spatially smoothed using a 6 mm full-width half maximum (FWHM) kernel. The first three volumes were removed to allow BOLD signal stabilization, after which white matter (WM) and cerebral spinal fluid (CSF) signals (obtained from fMRIprep before ICA-AROMA) were regressed out to remove residual physiological noise components (*fslglm*) (Beckmann et al., 2009). High-pass-filtering (200 s) was applied. For each functional scan, framewise displacement (FD) was calculated from low-pass filtered motion parameter time-series according to Gratton et al. (2020). Data from subjects with extreme motion (mean FD > 0.15 mm or > 120/400 frames exceeding a FD of 0.15 mm) were excluded from further analyses.

### 2.4 Connectivity matrix construction

The Brainnetome atlas was used to define 246 parcels (Fan et al., 2016) and fMRI signal time-series per participant per parcel were extracted. Cleaned fMRI time-series were then used to calculate Pearson's correlation connectivity matrices, resulting in a 246 x 246 connectivity matrix per participant, which was absolutized for further analyses. Furthermore, all matrices were z-scored and normalized to the total range of values per participant. Temporal signal-to-noise ratio (tSNR) maps were calculated per participant to calculate the lowest-quartile mask per age group per session. Parcels overlapping with the lowest-quartile of the tSNR maps with more than 70 % of voxels for more than 10 % of the participants were excluded from the connectivity matrices (Meijer et al., 2017). This was the case for the following regions: Orbital gyrus (OFG), superior temporal gyrus (STG), right middle temporal gyrus (R MTG), inferior temporal gyrus (ITG), right frontal gyrus (R FG), parahippocampal gyrus (PHG) and the left medioventral occipital cortex (L MVOC). These regions were subsequently removed from further analyses.

### 2.5 Obtaining graph measures

Connectivity strength (CS) and eigenvector centrality (EC) were calculated using the Brain Connectivity Toolbox (BCT) in Matlab (https://sites.google.com/site/bctnet/) from the normalized matrices (Rubinov and Sporns, 2010). Connectivity strength was used as a measure of the temporal correlations in the blood oxygenation level-dependent (BOLD) signals between our ROIs and the rest of the brain at rest. Secondly, eigenvector centrality was assessed as a measure of the extent to which our ROIs are connected to other highly connected regions, indicating the amount of influence of that ROI on the whole-brain network. We selected specific regions of interest (ROIs) involved in the mesolimbic dopaminergic pathway, namely the NAc, ventral striatum, amygdala, and the hippocampus (van Zessen et al., 2012). Additionally, we selected the insula, as it is part of a neural circuit involved in the prevention of overeating and ending feeding upon satiation (Wright et al., 2016). Finally, as part of the prefrontal cortex (PFC), the middle frontal gyrus (MFG) and superior frontal gyrus (SFG) were selected as they function as the projection zone of dopaminergic networks (Puig et al., 2014) and hereby are crucial for the contribution of dopaminergic signaling to conscious behavior (Kellendonk et al., 2006). All ROIs and their coordinates are displayed in Supplementary Table S1.

### 2.6 Statistical analysis

R Studio version 4.0.3 was used to perform all statistical analyses described below. All data were checked for normality and equality of variance, and outliers were removed (>3 SDs). In total, for the connectivity strength analysis, two data points had to be removed as outliers, and for the eigenvector centrality, three data points were removed. For both connectivity strength and eigenvector centrality, we performed an unpaired *t*-test comparing the low to the high intestinal ButCoA group in all ROIs. Age, BMI and Mean FD were tested as possible covariates, but were found not to be significant and thus not included in the analyses. A Bonferroni correction for multiple comparisons was applied, resulting into an adjusted alpha of 0.003125.

## 3 Results

### 3.1 Clinical characteristics

We could not acquire rs-fMRI data from three participants due to claustrophobia (N = 1) or technical failure on the day of the study visit (N = 2). In addition, rs-fMRI scans from five participants had to be excluded due to excess movement. Therefore, a total of 28 subjects with low ButCoA gene amount (mean 6.95 [3.89–10.01] percent ButCoA copies per 16S rRNA gene copy) and 24 subjects with high ButCoA gene amount (mean 372.03 [286.37–457.68] percent ButCoA copies per 16S rRNA gene copy) were included in this study. Clinical characteristics of the subjects per group (Table 1) showed no differences in baseline demographics. Importantly, the groups differed significantly in intestinal microbiota composition, as was analyzed via a machine learning classification model, reported in the main article (Wijdeveld et al., 2023). This analysis showed that the high ButCoA group was specifically characterized by higher abundance of various *Lachnospiraceae, Bacteroides* and *Agathobacter* species.

**Table 1:** Baseline characteristics of study participants (n = 52).

	Low ButCoA group	High ButCoA group	Statistic	p-value
	N = 28	N = 24		
**Age (years)**	53.32 ± 10.14	53.08 ± 10.14	−0.084	0.93
**Sex (N female (%))**	15 (53.57 %)	14 (58.33 %)	0.0042	0.95

**Height (cm)**	175.13 ± 9.70	175.43 ± 7.06	0.13	0.90
**Weight (kg)**	74.78 ± 13.53	77.13 ± 14.78	0.60	0.55
**BMI (kg/m^2^)**	24.35 ± 4.38	25.00 ± 4.34	0.54	0.60
**Body fat (%)**	25.97 ± 8.12	27.65 ± 9.24	0.69	0.49

**SBP (mmHg)**	119.78 ± 14.46	120.15 ± 16.36	0.086	0.93
**DBP (mmHg)**	74.87 ± 8.93	74.76 ± 8.30	−0.043	0.97
**Heartrate (bpm)**	60.57 ± 12.43	63.57 ± 8.59	1.02	0.32

**Fasted glucose (mmol/L)**	5.15 ± 0.52	5.21 ± 0.43	0.47	0.64
**Fasted insulin (pmol/L)**	28.74 ± 15.51	28.89 ± 14.39	0.036	0.97

**Total cholesterol (mmol/L)**	4.74 ± 0.95	4.80 ± 0.96	0.22	0.82
**HDL (mmol/L)**	1.53 ± 0.49	1.63 ± 0.37	0.85	0.41
**LDL (mmol/L)**	2.86 ± 0.94	2.80 ± 0.87	0.26	0.80
**Triglycerides (mmol/L)**	0.78 ± 0.45	0.84 ± 0.42	0.45	0.66

Tested with unpaired T-test. Pearson Chi-Square test had been performed to test for sex differences between both groups. For the paired *t*-test, the t-statistic, and for the chi-square test the X-squared statistic are reported. Numerical values are expressed as means ± standard deviations. No differences in characteristics were found between the groups. BMI = body mass index; bpm = beats per minute; mmol/L = millimole per liter; pmol/L = picomole per liter; mmHg = millimeter mercury.

### 3.2 Connectivity strength

**Fig. 1:**
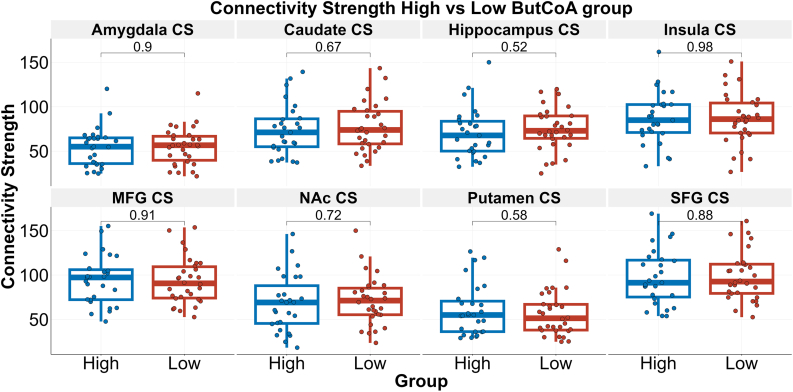
Connectivity strength in all ROIs per ButCoA group. Shown are boxplots and uncorrected p-values for each ROI. The high ButCoA group is depicted in blue; the low ButCoA group is depicted in red. ButCoA = butyryl-coenzyme A (CoA)-transferase; CS = connectivity strength; MFG = medial frontal gyrus; SFG = superior frontal gyrus; NAc = nucleus accumbens.

Connectivity strength for each ROI and differences between the groups are shown in Fig. 1. Whole-brain connectivity strength was 78.35 (69.99–86.71) in the low ButCoA group and 76.84 (67.07–86.61) in the high ButCoA group, and did not significantly differ between groups (p = 0.80). In addition, no differences in connectivity strength were observed between groups in any of the eight ROIs.

### 3.3 Eigenvector centrality

**Fig. 2:**
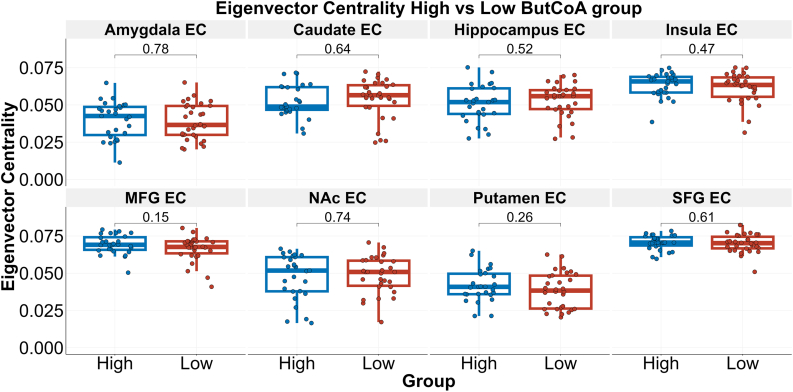
Eigenvector centrality in all ROIs per ButCoA group. Shown are boxplots and uncorrected p-values for each ROI. The high ButCoA group is depicted in blue; the low ButCoA group is depicted in red. ButCoA = butyryl-coenzyme A (CoA)-transferase; EC = eigenvector centrality; MFG = medial frontal gyrus; SFG = superior frontal gyrus; NAc = nucleus accumbens.

Mean eigenvector centrality for each ROI and differences between the groups are depicted in Fig. 2. Mean whole-brain eigenvector centrality was 0.058 (0.0576–0.0584) in the low ButCoA group and 0.058 (0.0578–0.0586) in the high ButCoA group, and did not significantly differ between groups (p = 0.54). In addition, no differences in eigenvector centrality were observed between groups in any of the eight ROIs.

## 4 Discussion

In this study, we compared functional connectivity of the mesolimbic dopaminergic network and relevant cortical areas affected by dopaminergic projections between individuals with extremely high and extremely low intestinal ButCoA gene amount. ButCoA gene amount reflects the intestinal acetate into butyrate converting activity, with high ButCoA amounts indicating both high intestinal acetate availability and high butyrate production (Diez-Gonzalez et al., 1999; Duncan et al., 2002). In contrast to our hypothesis, we found no differences in connectivity strength or eigenvector centrality for any of our ROIs between both groups. These findings do not support the presumed regulatory function of SCFAs in functional connectivity within the mesolimbic dopaminergic network.

We hypothesized that high ButCoA individuals would have altered connectivity and centrality of the mesolimbic pathway and its primary projection regions, as compared to low ButCoA individuals. This hypothesis was based on three lines of evidence. First, a link between physiological microbiota variations and resting state brain activity has previously been established by a study which demonstrated microbial alpha and beta diversity was related to resting state functional connectivity of the insula to the left frontal pole (Curtis et al., 2019). In addition, the abundance of specific bacterial genera was associated with default mode and executive control network connectivity (Kohn et al., 2021). Second, increased intestinal fermentation activity affects functional connectivity of specific networks in the brain, as was shown by a 4-week multi-strain probiotic intervention in humans (Bagga et al., 2019). This trial reported lower functional connectivity in the middle and superior frontal gyrus network, with increasing functional connectivity in the salience network. In addition, a study in healthy women showed that dietary supplementation with fermented milk increased midbrain resting state connectivity (Tillisch et al., 2013). Although this milk contained specific bacterial strains including *Bifidobacterium animalis* subsp *Lactis*, *Streptococcus thermophiles, Lactobacillus bulgaricus*, and *Lactococcus lactis* subsp *Lactis*, no significant change in microbiota composition was observed after the intervention. This raises important questions regarding the underlying mechanisms of action, suggesting that the observed changes in brain connectivity may not have been mediated through broad shifts in microbial community structure. Instead, transient metabolic effects, modulation of microbial gene expression, or localized host-microbe interactions at the mucosal interface—rather than compositional changes per se—may have contributed to the neurofunctional outcomes. Another 4-week probiotic intervention consisting of the same genera but different strains also induced significant changes in resting state functional connectivity, but partially in opposite direction (Rode et al., 2022). However, the absence of microbiota profiling before and after the intervention limits the ability to directly link these neural changes to specific alterations in gut microbial composition or activity. As such, the putative effects of the probiotic intervention on the gut microbiome remain speculative. Third, with regard to SCFAs, animal studies have linked increased peripheral acetate levels to appetite reduction, reflected by increased hypothalamic activity, modulated via reduction of hypothalamic AMPK activity (Frost et al., 2014). It has also been established in animal models that acetate administered through both intravenous (Perry et al., 2016) and colonic routes (Frost et al., 2014) is taken up by the brain and here can affect central appetite control via increased POMC and reduced AgRP expression in the hypothalamus.

The unique design of our study enabled us to study the link between acetate conversion into butyrate and resting state functional connectivity in healthy human participants in a cross-sectional design. A possible explanation for the absence of an association between ButCoA and functional connectivity is that we are not sure that the differences in intestinal butyrate turnover in our current study were linked to increased plasma acetate or butyrate concentrations, as we did not measure them due to their volatile nature. However, ButCoA is the most reliable proxy for long-term intestinal availability of acetate and butyrate. It is also possible that differences between groups might be present in brain regions that are direct targets of acetate, prior to activation of the mesolimbic pathways, such as the arcuate nucleus of the hypothalamus. However, since visualization of the hypothalamus is hampered by its location in the brain (De Silva et al., 2012), we did not include the hypothalamus in our ROIs. The absence of different network connectivity strength and centrality between the high and low ButCoA group may also be due to insufficient statistical power. However, we based our power calculations on previous fMRI studies by our group (ten Kulve et al., 2016; Doornweerd et al., 2018) and others (Stoeckel et al., 2008), addressing activity in comparable CNS circuits involved in satiety and reward regulation, suggesting that 27 participants per group was sufficient to detect BOLD signal differences. Moreover, the studies demonstrating the relationship between microbiota and resting state functional connectivity had similar numbers of participants, which sufficed to obtain significant results.

A more nuanced explanation for the absence of an association in our study is that the relation between microbiota and resting state connectivity may be mediated through other mechanisms, beyond local SCFA production. While SCFAs acetate and butyrate have been widely studied for their potential roles in modulating central nervous system activity, including appetite regulation and inflammation, it is increasingly evident that SCFA levels alone may not account for the full spectrum of microbiota–brain interactions. Specifically, local butyrate production may not directly translate to functional alterations in brain networks, either due to limited systemic absorption, inefficient signaling through vagal or endocrine pathways, or interindividual differences in receptor sensitivity and downstream signaling cascades. These null findings underscore the need to move beyond simplistic linear models of single-metabolite influence and toward more integrative, systems-level frameworks. Alternative microbiota-derived metabolites and pathways may play a more significant or synergistic role in shaping brain connectivity. For instance, microbiota-derived metabolites such as tryptophan catabolites (Agus et al., 2018), dopamine and other catecholamines (Asano et al., 2012), secondary bile acids (Ridlon et al., 2016), or bacterial cell wall components (e.g. lipopolysaccharide) (Fung et al., 2017) may also affect central signaling via immune activation, vagal stimulation, or neuroinflammation. Gut permeability and circulating cytokine levels could further modulate the microbiota-brain interface, introducing another layer of interindividual variability (Cryan and Dinan, 2012). An altered microbiota composition can lead to deficiencies in CNS dopaminergic circuitry, hereby influencing appetite regulation, although the exact mechanisms remain to be uncovered (Asano et al., 2012). Plausible modulating mechanisms include: reciprocal connections of the vagus nerve (Bravo et al., 2011), indirect effects via signaling molecules such as serotonin precursors, GABA, or improvement of epithelial barrier function (Kelly et al., 2015).

Furthermore, dopaminergic and other neuromodulatory systems may represent additional, underexplored avenues for gut–brain interaction. The gut microbiota plays a role in maintaining local and possibly systemic levels of dopamine and other catecholamines (Asano et al., 2012; Ge et al., 2018), which may influence central appetite regulation and reward circuitry. Dysbiosis has been associated with disruptions in dopaminergic signaling in animal models, though the mechanisms remain poorly defined. Possible routes of influence include the vagus nerve's bidirectional communication between intestines and the central nervous system (Bravo et al., 2011), paracrine signaling via serotonin and GABA precursors, or microbiota-mediated improvements in epithelial barrier function that alter systemic inflammation and neuromodulator availability (Kelly et al., 2015).

In light of these considerations, the null association between ButCoA levels and rs-FC observed in our study does not necessarily negate the potential role of the gut microbiota in modulating brain connectivity. Rather, it suggests that ButCoA expression alone may be an insufficient proxy for the functional influence of the intestinal ecosystem on the brain. Future research should consider adopting multi-omic and longitudinal designs to account for the dynamic and multi-faceted nature of gut-brain communication, including microbial gene expression, epigenetics, host inflammatory markers, proteomics, and individual differences in neurochemical and hormonal pathways. Such integrative approaches may better capture the complex, and possibly nonlinear, relationships that underlie microbiota–brain connectivity and ultimately inform individualized interventions.

In addition to these suggested alternate pathways affecting the gut-brain axis, future studies investigating the relationship between gut microbiota and resting-state functional connectivity should consider expanding their focus beyond brain regions traditionally associated with the mesolimbic dopaminergic system. While these regions are central to reward processing and have been frequently implicated in microbiota-brain signaling, other networks may also be sensitive to microbial influences. For instance, the anterior cingulate cortex is a key hub in the salience network and involved in interoceptive awareness and affective regulation—functions closely tied to gut-brain communication. Similarly, regions within the default mode network, such as the posterior cingulate cortex and medial prefrontal cortex, have been implicated in prior microbiota-intervention studies and may reflect broader changes in self-referential processing or mood-related states.

## 5 Conclusion

In conclusion, we did not observe a difference in network connectivity strength or centrality in brain regions within the mesolimbic dopaminergic network and cortical projection areas between people with extremely high and with extremely low intestinal ButCoA levels. Our findings do not support a link between intestinal butyrate turnover and resting state functional connectivity in dopaminergic regions of the brain. We suggest that other mechanisms, beyond local SCFA production, may underlie the intricate relation between intestinal microbiota activity and resting state functional connectivity. Future studies should investigate these alternative pathways and also consider examining additional brain regions that may be modulated by gut microbial activity beyond the dopaminergic system.

## CRediT authorship contribution statement

**Madelief Wijdeveld:** Writing – original draft, Methodology, Formal analysis, Data curation. **Anouk Schrantee:** Writing – review & editing, Supervision, Software, Methodology, Formal analysis, Conceptualization. **Júlia Tolra Azor:** Writing – original draft, Data curation. **Francesca van Baarzel:** Writing – review & editing, Visualization, Formal analysis. **Eelco van Duinkerken:** Writing – review & editing, Formal analysis, Conceptualization. **Max Nieuwdorp:** Supervision, Project administration, Funding acquisition. **Richard G. Ijzerman:** Writing – review & editing, Supervision, Resources, Investigation, Conceptualization.

## Funding

M. Wijdeveld is supported by the AMC MD/PhD fellowship 2017. M. Nieuwdorp is supported by a ZONMW VICI grant 2020 (09150182010020). A. Schrantee is supported by a 10.13039/501100003246Dutch Research Council Veni grant (016.196.153).

## Declaration of competing interest

The authors declare the following financial interests/personal relationships which may be considered as potential competing interests: M.N. is in the Scientific Advisory Board of Caelus Pharmaceuticals, in the Netherlands, however this is not relevant for the contents of the current manuscript. The other authors declare that they have no known competing financial interests or personal relationships that could have appeared to influence the work reported in this paper.

## Data Availability

The 16S rRNA gene amplicon raw sequence data and associated metadata have been deposited at the European Genome-phenome Archive under accession code EGAD00001004106 (https://ega-archive.org/datasets). The HELIUS data are all owned by the Amsterdam UMC, location AMC in Amsterdam, the Netherlands. Any researcher can request the data by submitting a proposal as outlined at http://www.heliusstudy.nl/. The study data that support the findings of this study are available on request from the corresponding author.
