# Supplementary material for: Intestinal short-chain fatty acid turnover is not associated with resting state functional connectivity in mesolimbic dopaminergic network in healthy adults
Source: Neuroimage Rep. 2025 Aug 25;5(3):100285. doi: 10.1016/j.ynirp.2025.100285 (PMC12398794; doi:10.1016/j.ynirp.2025.100285)
Supplement: Multimedia component 1 [file mmc1.docx]

**SUPPLEMENTARY METHODS**


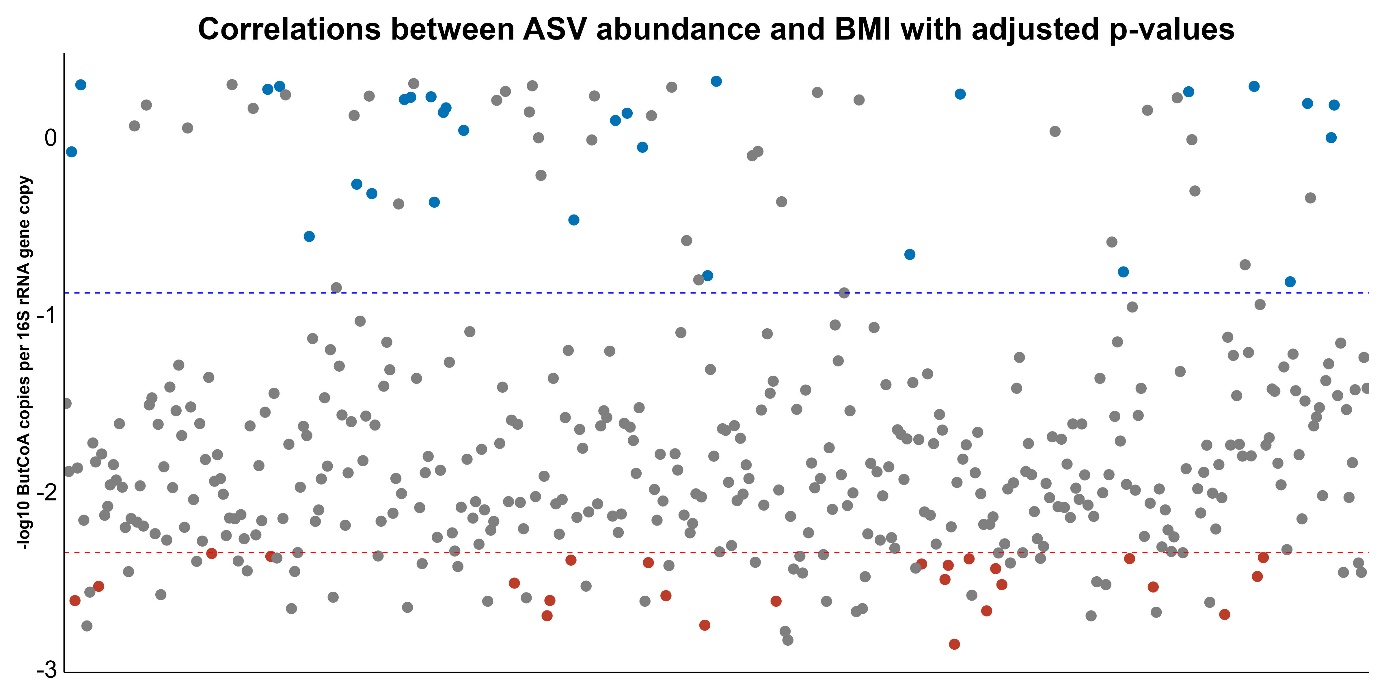


**Supplementary Figure S1.** Selection of participants based on 439 Dutch origin participants, of whom fecal material was collected and qPCR was performed to measure the relative concentration of the ButCoA gene. Each dot represents one participant. Y-axis represents the inverted 10log of the total count of BUTCoA copies per 16S gene copy. The red line indicates the cut-off for the lower 15% of BUTCoA gene copies, the blue line indicates the cut-off for the upper 15%. Each red dot represents a participant who was included in the low BUTCoA group, each blue dot represents a participant who was included in the high BUTCoA group.

| **Supplementary Table S1. Human Brainnetome atlas region numbers per selected ROI** | | | | | |
| --- | --- | --- | --- | --- | --- |
|  |  |  |  |  |  |
| **Brainnetome Region of Interest** | **Brainnetome region numbers left** | **Brainnetome region numbers right** | **Anatomical and modified Cyto-architectonic descriptions** | **Left MNI coordinates (x,y,z)** | **Right MNI coordinates (x,y,z)** |
| **Amygdala** | | | | | |
| Amyg_L(R)_2_1 | 211 | 212 | mAmyg, medial amygdala | -19, -2, -20 | 19, -2, -19 |
| Amyg_L(R)_2_2 | 213 | 214 | lAmyg, lateral amygdala | -27, -4, -20 | 28, -3, -20 |
| **Hippocampus** | | | | | |
| Hipp_L(R)_2_1 | 215 | 216 | rHipp, rostral hippocampus | -22, -14, -19 | 22, -12, -20 |
| Hipp_L(R)_2_2 | 217 | 218 | cHipp, caudal hippocampus | -28, -30, -10 | 29, -27, -10 |
| **Superior frontal gyrus** | | | | | |
| SFG_L(R)_7_1 | 1 | 2 | A8m, medial area 8 | -5 ,15, 54 | 7, 16, 54 |
| SFG_L(R)_7_2 | 3 | 4 | A8dl, dorsolateral area 8 | -18, 24, 53 | 22, 26, 51 |
| SFG_L(R)_7_3 | 5 | 6 | A9l, lateral area 9 | -11, 49, 40 | 13, 48, 40 |
| SFG_L(R)_7_4 | 7 | 8 | A6dl, dorsolateral area 6 | -18, -1, 65 | 20, 4, 64 |
| SFG_L(R)_7_5 | 9 | 10 | A6m, medial area 6 | -6, -5, 58 | 7, -4, 60 |
| SFG_L(R)_7_6 | 11 | 12 | A9m,medial area 9 | -5, 36, 38 | 6, 38, 35 |
| SFG_L(R)_7_7 | 13 | 14 | A10m, medial area 10 | -8, 56, 15 | 8, 58, 13 |
| **Middle frontal gyrus** | | | | | |
| MFG_L(R)_7_1 | 15 | 16 | A9/46d, dorsal area 9/46 | -27, 43, 31 | 30, 37, 36 |
| MFG_L(R)_7_2 | 17 | 18 | IFJ, inferior frontal junction | -42, 13, 36 | 42, 11, 39 |
| MFG_L(R)_7_3 | 19 | 20 | A46, area 46 | -28, 56, 12 | 28, 55, 17 |
| MFG_L(R)_7_4 | 21 | 22 | A9/46v, ventral area 9/46 | -41, 41, 16 | 42, 44, 14 |
| MFG_L(R)_7_5 | 23 | 24 | A8vl, ventrolateral area 8 | -33, 23, 45 | 42, 27, 39 |
| MFG_L(R)_7_6 | 25 | 26 | A6vl, ventrolateral area 6 | -32, 4, 55 | 34, 8, 54 |
| MFG_L(R)_7_7 | 27 | 28 | A10l, lateral area10 | -26, 60, -6 | 25, 61, -4 |
| **Insula** | | | | | |
| INS_L(R)_6_1 | 163 | 164 | G, hypergranular insula | -36, -20, 10 | 37, -18, 8 |
| INS_L(R)_6_2 | 165 | 166 | vIa, ventral agranular insula | -32, 14, -13 | 33, 14, -13 |
| INS_L(R)_6_3 | 167 | 168 | dIa, dorsal agranular insula | -34, 18, 1 | 36, 18, 1 |
| INS_L(R)_6_4 | 169 | 170 | vId/vIg, ventral dysgranular and granular insula | -38, -4, -9 | 39, -2, -9 |
| INS_L(R)_6_5 | 171 | 172 | dIg, dorsal granular insula | -38, -8, 8 | 39, -7, 8 |
| INS_L(R)_6_6 | 173 | 174 | dId, dorsal dysgranular insula | -38, 5, 5 | 38, 5, 5 |
| **Caudate nucleus** | | | | | |
| BG_L(R)_6_1 | 219 | 220 | vCa, ventral caudate | -12, 14, 0 | 15, 14, -2 |
| BG_L(R)_6_5 | 227 | 228 | dCa, dorsal caudate | -14, 2, 16 | 14, 5, 14 |
| **Putamen** | | | | | |
| BG_L(R)_6_4 | 225 | 226 | vmPu, ventromedial putamen | -23, 7, -4 | 22, 8, -1 |
| BG_L(R)_6_6 | 229 | 230 | dlPu, dorsolateral putamen | -28, -5, 2 | 29, -3, 1 |
| **Nucleus accumbens** | | | | | |
| BG_L(R)_6_3 | 223 | 224 | NAC, nucleus accumbens | -17, 3, -9 | 15, 8, -9 |
|  | | | | | |
| Eight regions from the human Brainnetome Atlas selected as primary regions of interest (ROIs), including region numbers, architectonic description and coordinates. MNI = Montreal Neurological Institute. | | | | | |

**TABLE AND FIGURE LEGENDS**

**Supplementary Figure S1.** Selection of participants based on 439 Dutch origin participants, of whom fecal material was collected and qPCR was performed to measure the relative concentration of the ButCoA gene. Each dot represents one participant. Y-axis represents the inverted 10log of the total count of BUTCoA copies per 16S gene copy. The red line indicates the cut-off for the lower 15% of BUTCoA gene copies, the blue line indicates the cut-off for the upper 15%. Each red dot represents a participant who was included in the low BUTCoA group, each blue dot represents a participant who was included in the high BUTCoA group.

**Supplementary Table S1.** Eight regions from the human Brainnetome Atlas selected as primary regions of interest (ROIs), including region numbers, architectonic description and coordinates. MNI = Montreal Neurological Institute.
